# Potential risk factors for early and late dental implant failure: a retrospective clinical study on 9080 implants

**DOI:** 10.1186/s40729-020-00276-w

**Published:** 2020-11-30

**Authors:** Henning Staedt, Martin Rossa, Karl Martin Lehmann, Bilal Al-Nawas, Peer W. Kämmerer, Diana Heimes

**Affiliations:** 1 Private Practice and Department of Prosthodontics and Materials Science, University Medical Center Rostock, Strempelstraße 13, 18057 Rostock, Germany; 2 Private Practice, Dr. Rossa und Kollegen, Mundenheimer Str. 251, Ludwigshafen, 67061 Germany; 3 Department of Prosthetic Dentistry, University Medical Center Mainz, Augustusplatz 2, 55131 Mainz, Germany; 4 Department of Oral and Maxillofacial Surgery, University Medical Center Mainz, Augustusplatz 2, 55131 Mainz, Germany

**Keywords:** Dental implants, Early dental implant failure, Late dental implant failure, Human, Cohort study, Risk factor

## Abstract

**Background:**

The aim of this study was to analyze potential risk factors for early and late dental implant failure (DIF) in a clinical cohort trial.

In a private practice, 9080 implants were inserted during a period of 10 years. In case of DIF, data were classified into early and late DIF and compared to each other in regard of gender, age, site of implantation, implant geometry, and patients’ systemic diseases.

**Results:**

Three hundred fifty-one implants failed within the observation period (survival rate: 96.13%). Early DIF occurred in 293 implants (83.48%) compared to late DIF in 58 implants (16.52%). Significant earlier DIF was seen in the mandible (OR = 3.729, *p* < 0.001)—especially in the posterior area—and in younger patients (*p* = 0.017), whereas an increased likelihood of late DIF was associated with maxillary implants (OR = 3.729, *p* < 0.001) and older patients.

**Conclusions:**

Early DIF is about twice as common as late DIF. Main risk factors for early DIF are implant location in the (posterior) mandible as well as younger age. On contrary, late DIF is rather associated with older patients, cancellous bone quality, and longer implants.

## Background

The insertion of osseointegrated dental implants is a reliable treatment option for rehabilitating fully or partially edentulous patients. Despite high success rates, the individual optimization of treatment protocols is crucial for prognosis and patients’ satisfaction and analysis of potential risk factors for dental implant failure is an issue of increasing interest. Over an observation period of 10 years, a survival rate of 85–95% can be estimated [1]. In 5%, the absence of primary implant integration results in implant failure [2] and an intra-individual accumulation of implant losses might imply the existence of specific risk factors for dental implant failure (DIF) [3, 4]. DIF can be divided into early and late events. Early DIF is associated with impaired bone healing. In case of insufficient bone-implant contact, fibrous scar formation leads to a loosening of the bone-implant interface [2, 5–10]. After a latency of 6 months, late DIF occurs [5, 6, 11–14].

The respective risk factors can be subdivided into iatrogenic, material-associated, and patient-related factors [15]. Side effects during surgery include heat-induced necrosis, poor primary stability, and incorrect positioning [9, 16, 17]. The implants’ geometry—including the implant’s dimensions and its macro-design—as well as the type of prosthetic treatment does affect loading distribution and in consequence the dental implants’ survival rate [18–20]. Local risk factors include significant plaque accumulation, gingivitis, tight implant-tooth contact [9], bone quality and quantity [21–24], poor oral hygiene, periodontal disorders, and chronic occlusal trauma [25]. Also, systemic factors like xerostomia, osteoporosis, cardiovascular diseases, and diabetes mellitus are reported to influence the patients’ wound-healing capability [5, 15, 20, 26–29].

The purpose of this study was to evaluate potential risk factors (age, gender, site of implant placement, implant geometry, systemic disease) for early and late DIF in a retrospective cohort analysis.

## Methods

### Study design

Patients, who received dental implants for different reasons within a defined period of 10 years (2002-2012) participated in the present study. The patients included are a subsample of all patients treated within the dental practice. Two hundred sixty-six patients, with a total of 351 implants that failed within the observation period, were analyzed retrospectively regarding the implants’ survival time and conditions. The patients’ medical records were checked for potential risk factors and, in case of further questions, the patients or their family doctors were consulted. A total of 9080 cases fitted the inclusion criteria: Homogeneity was ensured by using the same implant system (helical HiTec Tapered Self Thread implant, Hi-Tec Implants, Herzlia, Israel) inserted by the same dentists (S.H. and R.M.) in one practice. HiTec Tapered Self Thread implants are titanium implants designed with a tapered body and v-shaped threads. The apex is dome shaped and contains grooves. A hexagonal internal connection is placed within the straight head [30].

Special interest was directed toward the analysis of implant failures. Implant failure was defined in case of high implant mobility and/or pain or infection (including peri-implant radiolucency). Implants showing those signs were removed. Besides, a lost implant was defined to be an implant failure as well. The data was pseudonymized—therefore, no approval by the ethics commission was needed.

### Surgery

The implants were placed under aseptic conditions after professional tooth cleaning and—if necessary—periodontal treatments according to the manufacturer’s specifications (see Table 1). Before preparing the implantation site, augmentation techniques were performed depending on the patients’ characteristics. Teeth were removed and the bone was allowed to heal for 8 to 16 weeks. The protocol was performed depending on the patient’s health, clinical situation, and bone quality and quantity. The basic surgical protocol was to place implants and abutments at once (1-stage) when treating patients in the—especially maxillary—front tooth area (*n* = 81). A 2-staged surgery was the basic protocol for posteriorly placed implants (*n* = 230). Prosthetic loading was conducted according to surgical standards (hygiene, precision, soft tissue management) and after a latency of either three to 4 months in implants placed within the mandible or 4 to 6 months in implants inserted within the maxilla. After the insertion of the two-piece implants, titanium abutments with fixed partial dentures were utilized. It was taken care that static and dynamic occlusion was checked intensively.

**Table 1:** Contingency analysis

	Pearson’s chi-square (***χ***^**2**^)	***P*** value	Phi (***Φ***) contingency coefficient	Cramers ***V***
**Age**	5.743	**0.017**	0.147	
**Gender**	0.014	0.904	0.007	
**Jaw**	13.358	**0.001**	0.224	
**Location**	6.635	0.383	0.053	
**Length**	13.554	**0.004**		0.197
**Diameter**	2.510	0.474		0.085
**CVD**	1.355	0.244	0.071	
**Diabetes mellitus**	0.707	0.400	0.052	
**Comorbidity**	4.741	**0.029**	0.134	

The table shows the computation of Pearson’s chi-square (*χ*^2^) test, which was performed to check the correlation between the variables. As chi-square test lacks standardization, correlative measures like Phi (*Φ*) contingency coefficient (four fields table) and Cramers *V* (≥ 4 cases) were used to demonstrate the strength of association between the groups. A value of ≥ 0.1 was defined as a low association, a value of ≥ 0.3 up to < 0.5 as medium strength of association, and a value ≥ 0.5 as a strong association between the groups. A *p* value ≤ 0.05 was termed significant

### Follow-up

All patients were followed up by the dentist in charge for a mean of 5.42 years (min. = 1 month, max. = 120 months, SD = 20.76 months) or until the implant failed/was removed. Patients who missed the follow-up appointments or left the practice within a period of 2 years were excluded from the study. Radiographs were taken preoperatively and directly after surgery. Clinical evaluations including soft tissue quality, healing at the implant site, implant stability, and periodontal status were conducted after 6 months. One year after surgery, radiographs were taken to evaluate bone resorption and quality. In case of adequate healing and implant stability, clinical evaluations were done every 6 months; if there was any sign of pain, infection, healing delay, or implant instability, recall intervals were shorter.

### Parameters

Dental implant failure (DIF) was recorded and subdivided into groups of early and late events. Early DIF was defined as high implant mobility and/or pain or infection (including peri-implant radiolucency) within a period of 6 months after implantation. Besides, a lost implant within this period was defined to be an early implant failure as well. The occurrence of pathological radiological or clinical characteristics and the loss of an implant beginning after a latency of 6 months was termed as late DIF. The patients’ gender and age as well as the implant location and geometric features (diameter and length) were collected and ranked. Furthermore, the occurrence of systemic diseases was analyzed as potential risk factors.

### Statistics

In addition to the calculated overall implant survival (as ratio between implants in situ and failed implants), the incidence of potential risk factors was compared between the group with early and late DIF. A differentiation between risk factors on patient level (each patient as the statistical unit with patients presenting or not presenting implant failure) and implant level was done. As the data contained nominal variables, Pearson’s chi-Square (*χ*^2^) test was performed to check the correlation between the variables. As chi-square test lacks standardization, correlative measures like Phi (*Φ*) contingency coefficient (four fields table) and Cramers *V* (≥ 4 cases) were used to demonstrate the strength of association between the groups. A value of ≥ 0.1 was defined as a low association, a value of ≥0 .3 up to < 0.5 as medium strength of association, and a value ≥ 0.5 as a strong association between the groups. Metric variables were analyzed using the binary and multivariate logistic regression analysis and categorized regarding their odds ratio (OR). The data is displayed as OR with a confidence interval (CI). The statistical analyses were performed using SPSS version 24 for Windows (IBM, Armonk, New York). A *p* value ≤ 0.05 was termed significant.

## Results

### Descriptive analysis

**Fig. 1:**
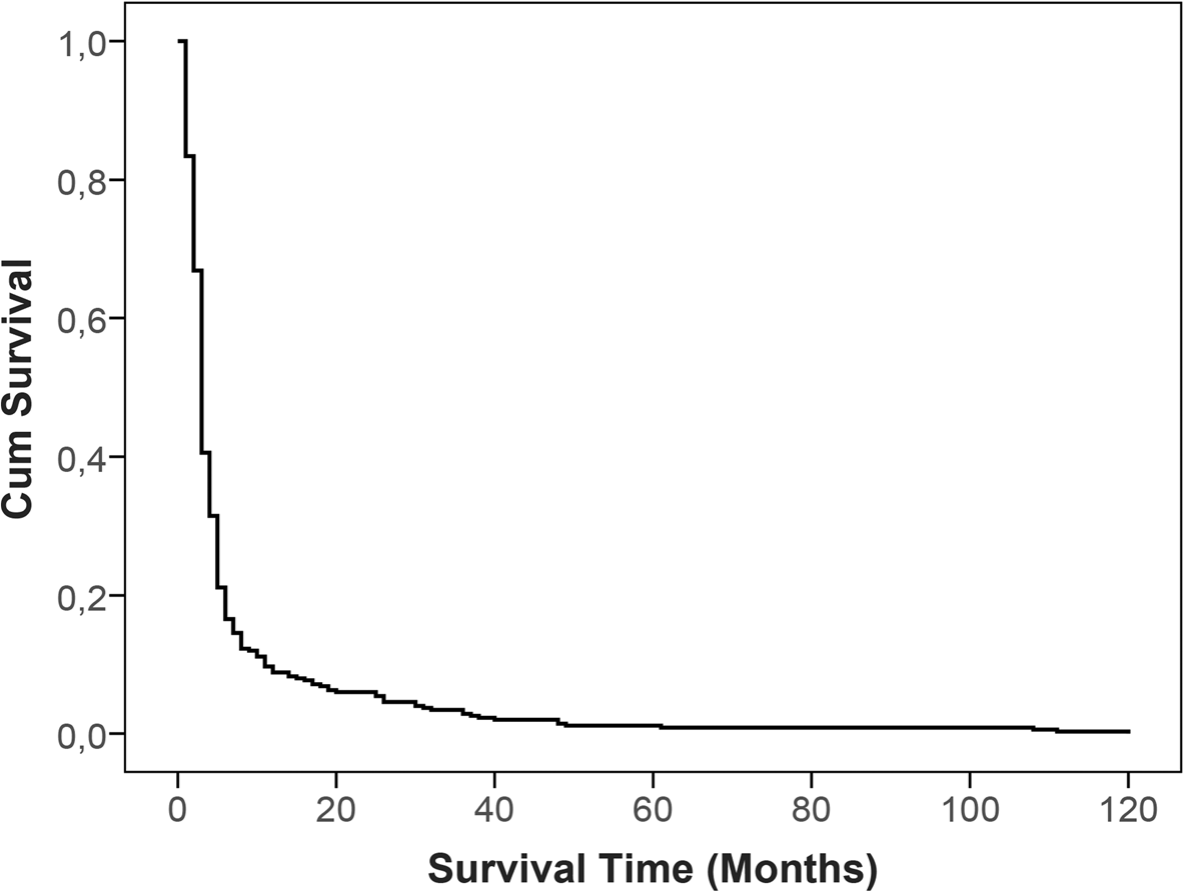
Cumulative survival analysis. The figure shows a Kaplan Meier curve which displays the overall implant failure over time

Within this study, 9080 dental implants were analyzed regarding their survival time. Patients who received dental implants within the defined period of time were followed up for a mean of 5.42 years (min. = 1 month, max. = 120 months, SD = 20.76 months) or until the implant failed/was removed. In total, 351/9080 implants failed among 266 patients. This corresponds to a survival rate of 96.13% (survival analysis see Fig. 1). Early dental implant failure occurred with a rate of 83.48% (*n* = 293), whereas late failure exhibited a 16.52% rate of occurrence (*n* = 58). One implant/patient was lost in 76.3% of cases (*n* = 203), whereas 23.7% of patients showed several implant losses.

### Age

**Fig. 2:**
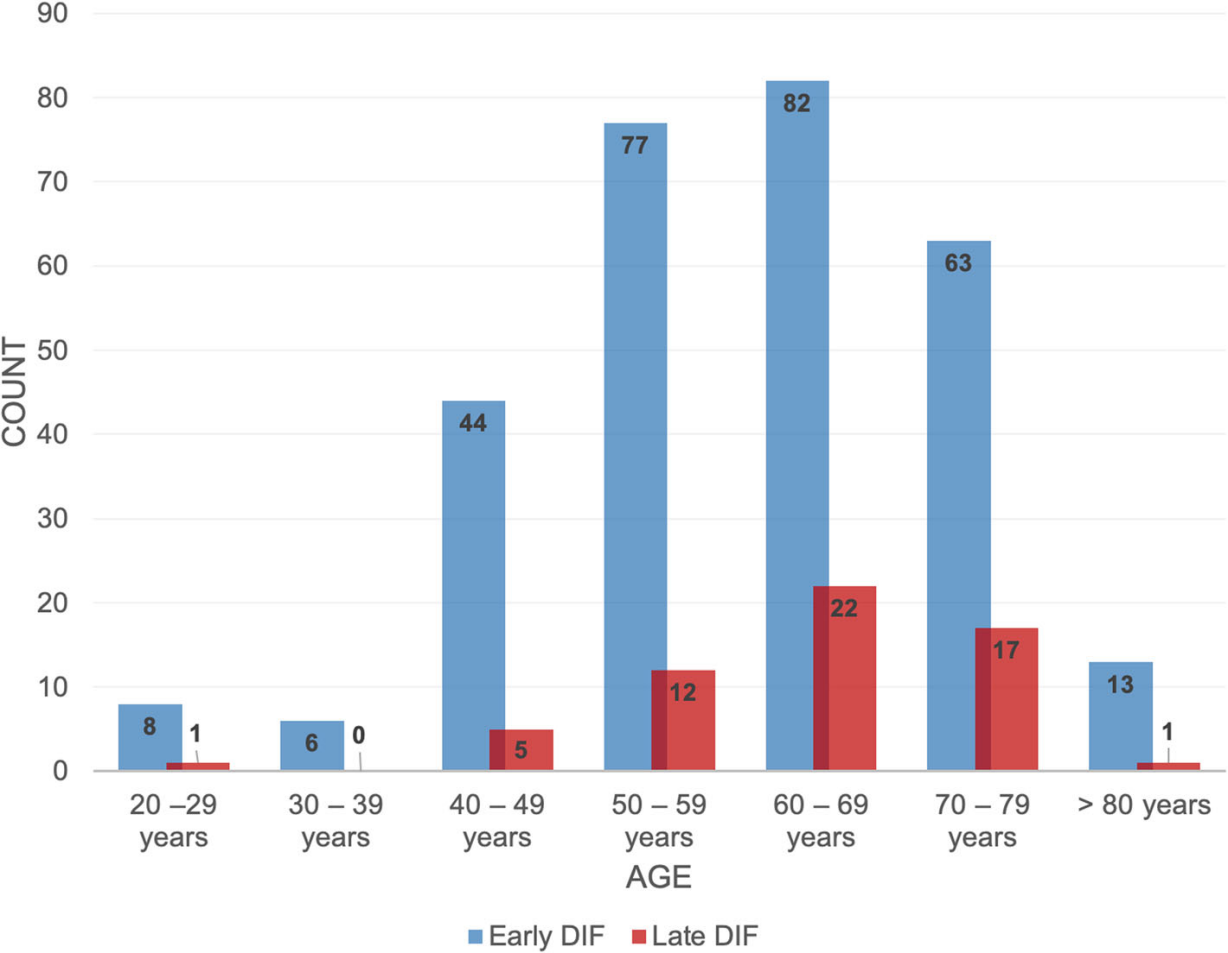
Frequency of early and late dental implant failure (DIF) among patients of different age

The total study population showed a mean age of 61.5 years (min. = 21, max. = 88, SD = 20.5). Both early and late DIF occurred most in patients of 60 to 70 years (see Fig. 2). The contingency analysis could display a statistically significant correlation between patients younger and older than 60 years, and the measured event (*χ*^2^ = 5.743, *p* = 0.017, *Φ* = 0.147).

### Gender

Males showed a higher incidence (*n* = 142, 56.7%) of implant failures than females (*n* = 124, 43.3%) without significant differences between groups. Even if early and late DIF accumulated among male patients (male patients early DIF: *n* = 116, 43.61% and late DIF: *n* = 26, 9.77%; female patients early DIF: *n* = 102, 38.35%, late DIF *n* = 22, 8.27%), no statistically significant correlation between the patients’ gender and the measured event was seen (*χ*^2^ = 0.014, *p* = 0.904, *Φ* = 0.007).

### Implant site location

A total of 178 implants (50.7%) in 136 patients inserted in the maxilla and 173 implants (49.3%) in 130 patients inserted in the mandible failed within the observation period. Early failure of implants inserted into the mandible was seen in 118 patients (44.36%), whereas maxillary placed implants failed in 100 patients (37.59%). Late DIF occurred thrice as often in the maxilla (*n* = 36, 13.53% versus *n* = 12, 4.51% in the mandible). The contingency analysis displayed a statistically significant correlation (*χ*^2^ = 13.358, *p* < 0.001) with a medium strength of association (*Φ* = 0.224).

Implants located in the frontal area (central incisor to canine) failed in 92 patients (34.59%), implants inserted in the posterior area of the jaw (1st premolar to 2nd molar) in 174 patients (65.41%). Early DIF occurred more often in patients with posteriorly placed implants with a frequency of 52.63% (*n* = 140) in comparison to implants located in the front tooth area (*n* = 78, 29.32%). In accordance, late DIF was more frequent in patients with posteriorly placed implants (front: *n* = 14, 5.26% and posterior: *n* = 34, 12.78%). Here, the contingency analysis displayed no statistically significant correlation (*χ*^2^ = 6.635, *p* = 0.383, *Φ* = 0.053) between the groups.

### Implant geometry

**Fig. 3:**
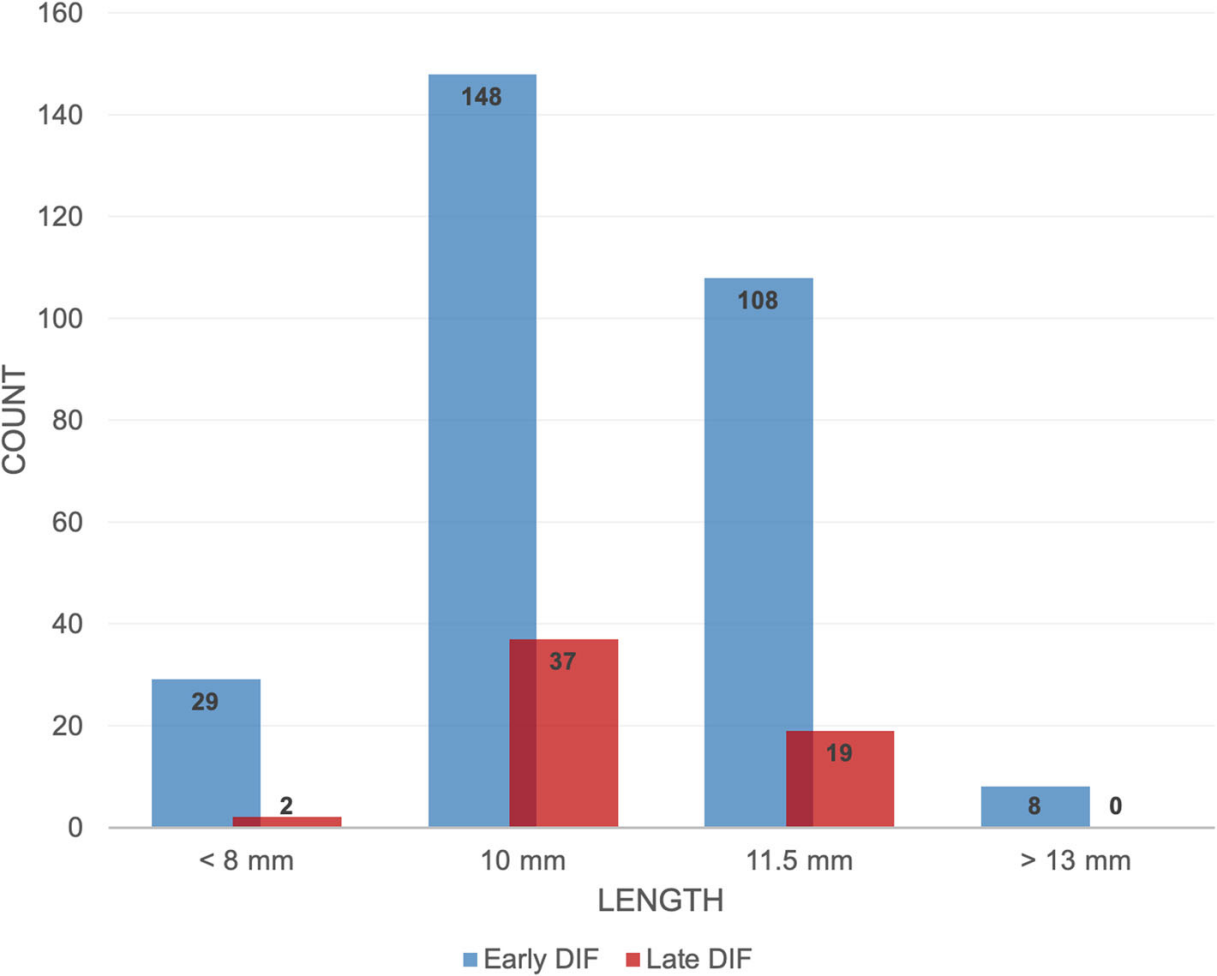
Frequency of early and late dental implant failure among implants of different length. Early DIF was frequent in implants of 10 mm (*n* = 134, 38.2%), whereas late dental implant failure showed high incidences in implants measuring 10 mm (*n* = 51, 14.5%) and 11.5 mm (*n* = 47, 13.4%) in length

**Fig. 4:**
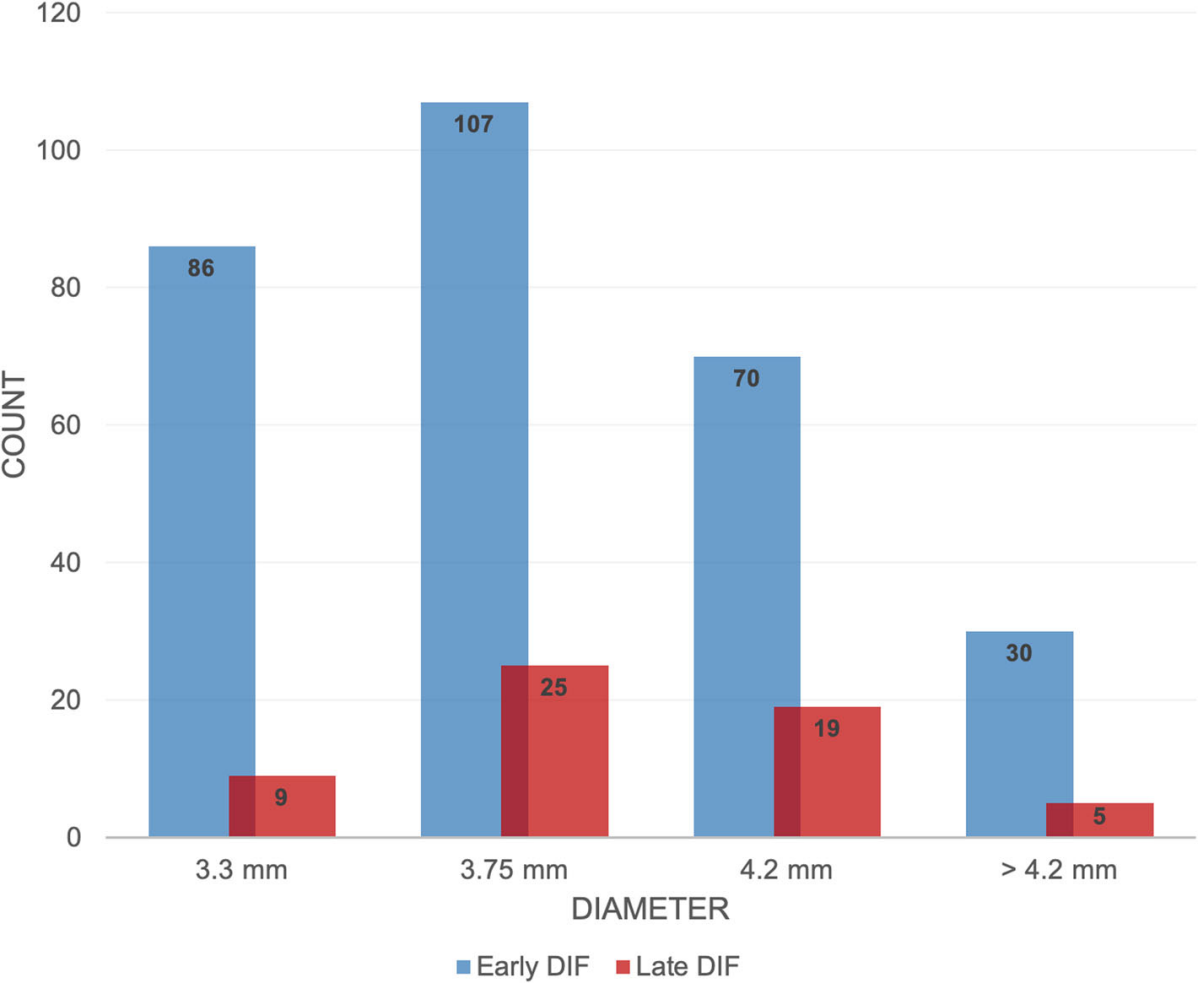
Frequency of early and late dental implant failure among implants of different diameter. Groups of different diameters displayed no significant correlation (*χ*^2^ = 2.510, *p* = 0.474, Cramers *V* = 0.085). Implants of smaller diameter showed higher frequencies of early events (3.3 mm: *n* = 72, 20.5%; 3.5 mm: *n* = 88, 25.1%; 4.2 mm: *n* = 62, 17.7%) and a maximum frequency of late losses in implants of 3.75 mm (*n* = 44, 12.5%) with no statistically significant correlation

A total of 9080 implants were inserted during the observation period. Ten percent (*n* = 908) had a length of 8 mm and smaller, 53% measured 10 mm (*n* = 4812), 35% 1.5 mm (*n* = 3178), and 2% of implants showed lengths of 13 mm and larger (*n* = 182). In general, an accumulation of DIF could be observed for implants measuring 10 mm in length (failed ratio: *n* = 185, 52.7%; total ratio: 31/4812), and 3.75 mm in diameter (failed ratio: *n* = 132, 37.6%; total ratio: 132/3360). Two thousand five hundred forty-two implants of 3.3 mm in diameter were inserted during the observation period (28%). Implants of 3.75 mm were inserted more frequently (*n* = 3360, 37%) than implants of 4.2 mm (*n* = 2270, 25%) and lager (*n* = 908, 10%). Early DIF occurred more often in implants measuring 10 mm (*n* = 134, 38.2%) in comparison to both, smaller and larger implants. Furthermore, a high frequency of late dental implant failure could be recorded in implants of both, 10 mm (*n* = 51, 14.5%) and 11.5 mm (*n* = 47, 13.4%) in length (see Fig. 3). The contingency analysis showed a statistically significant correlation (*χ*^2^ = 13.554, *p* = 0.004) and a medium strength of association (Cramers *V* = 0.197) between implant length and dental implant failure. In contrast, groups of different diameters displayed no significant correlation (*χ*^2^ = 2.510, *p* = 0.474, Cramers *V* = 0.085) with a greater incidence of early events in implants of smaller diameter (3.3 mm: *n* = 72, 20.5%; 3.5 mm: *n* = 88, 25.1%; 4.2 mm: *n* = 62, 17.7%) and a maximum frequency of late losses in implants of 3.75 mm (*n* = 44, 12.5%) (see Fig. 4).

### Systemic diseases

One hundred fifty-three patients were suffering from cardiovascular diseases (CVD; 57.52%), 7.52% (*n* = 20) from diabetes mellitus, and 12 patients (4.51%) showed a comorbidity of both cardiovascular diseases and diabetes mellitus.

#### Cardiovascular disease

In 89 (33.46%) patients suffering from CVD, implants failed early, whereas late DIF happened only in 24 patients (9.02%). The contingency analysis could not show a significant correlation (*χ*^2^ = 1.355, *p* = 0.244, *Φ* = 0.071) between CVD and DIF between both groups.

#### Diabetes mellitus

Fifteen patients (5.64%) suffering from diabetes mellitus showed an early and 5 patients (1.88%) a late event without significant correlation (*χ*^2^ = 0.707, *p* = 0.400, *Φ* = 0.052) between diabetes mellitus and both, early and late DIF.

#### Comorbidity

Analyzing a comorbidity of CVD and diabetes mellitus, 7 patients with early (2.63%) and 5 patients (1.88%) with late DIF were recorded. The contingency analysis showed a statistically significant correlation (*χ*^2^ = 4.741, *p* = 0.029) with a medium strength of association (*Φ* = 0.134) between a comorbidity and DIF in both groups.

### Early and late DIF: logistic regression analysis

A logistic regression analysis was performed to ascertain the effects of the variables analyzed within this study on early and late DIF. First, a binary logistic regression analysis was performed for the independent variables (see Table 2); thereafter, a multivariate logistic regression analysis was needed to ascertain the effects of each factor (see Table 3). The model for patients related factors (gender, age, jaw, CVD, diabetes mellitus) correctly classified 83.5% of cases with Nagelkerkes’ *R*^2^ of 0.111, which corresponds to a moderate explanatory power [31]. Analyzing the patient’s gender and age, the regression analysis did not show different probabilities for early or late events between the groups. Maxillary implants showed a reduced likelihood (OR = 0.260; *p* ˂ 0.001; CI, 0.135–0.501) for early DIF, whereas they were 3.849 times as much as likely to exhibit a late event (OR = 3.849; *p* ˂ 0.001; CI, 1.995–7.425) compared to implants placed in the mandible. Furthermore, the probabilities for early and late events did not differ between implants located within the anterior or posterior area of the jaw. Neither for diabetes nor for CVD associations with early and late DIF could be seen when comparing to healthy patients. The model for implant-related factors (length, diameter) correctly classified 63.5% of cases with Nagelkerkes’ *R*^2^ of 0.010, which corresponds to a low explanatory power. Implants of different lengths or diameter could not be associated with an increased likelihood of either early or late DIF.

**Table 2:** Binary logistic regression analysis

	Reference value	SE	Wald	***P*** value	OR late DIF	CI late DIF	OR early DIF	CI early DIF
**Age**	20-29 years	0.119	2.835	0.092	1.223	0.968-1.545	0.818	0.647-1.033
**Gender**	Female	0.289	0.066	0.798	0.929	0.527-1.637	1.077	0.611-1.898
**Jaw**	Maxilla	0.328	16.054	**< 0.001**	0.268	0.141-0.511	3.729	1.959-7.100
**Location**	Front	0.308	0.794	0.373	1.316	0.719-2.410	0.760	0.415-1.391
**Length**	< 8 mm	0.217	0.107	0.744	0.932	0.609-1.425	1.073	0.702-1.642
**Diameter**	3.3 mm	0.150	2.047	0.153	1.240	0.923-1.666	0.806	0.600-1.083
**CVD**	No CVD	0.036	1.490	0.222	1.045	0.975-1.121	0.957	0.892-1.027
**Diabetes mellitus**	No diabetes	0.454	0.723	0.395	1.471	0.604-3.583	0.680	0.279-1.655
**Comorbidity**	No comorbidity	0.474	2.855	0.091	2.228	0.880-5.645	0.449	0.177-1.1.37

*Abbreviations*: *SE* Standard error, *OR* Odds ratio, *CI* Confidence interval, *DIF* Dental implant failure

**Table 3:** Multivariate logistic regression analysis

	Reference value	SE	Wald	***P*** value	OR late DIF	CI late DIF	OR early DIF	CI early DIF
**Age**	20-29 years	0.119	2.835	0.092	1.223	0.968-1.545	0.818	0.647-1.033
**Gender**	Female	0.289	0.066	0.798	0.929	0.527-1.637	1.077	0.611-1.898
**Jaw**	Maxilla	0.328	16.054	**< 0.001**	0.268	0.141-0.511	3.729	1.959-7.100
**Location**	Front	0.308	0.794	0.373	1.316	0.719-2.410	0.760	0.415-1.391
**Length**	< 8 mm	0.217	0.107	0.744	0.932	0.609-1.425	1.073	0.702-1.642
**Diameter**	3.3 mm	0.150	2.047	0.153	1.240	0.923-1.666	0.806	0.600-1.083
**CVD**	No CVD	0.036	1.490	0.222	1.045	0.975-1.121	0.957	0.892-1.027
**Diabetes mellitus**	No diabetes	0.454	0.723	0.395	1.471	0.604-3.583	0.680	0.279-1.655
**Comorbidity**	No	0.474	2.855	0.091	2.228	0.880-5.645	0.449	0.177-1.1.37

*Abbreviations*: *SE* Standard error, *OR* Odds ratio, *CI* Confidence interval, *DIF* Dental implant failure

## Discussion

The aim of the study was an investigation of potential risk factors for early and late dental implant failure (DIF) by including both patient and implant-related factors. Despite low failure rates [1, 32], the evaluation of potential risk factors—especially regarding their impact on the moment of implant loss—is crucial for receiving a sustainable and secure long-term provision. Limited numbers of large studies examining risk factors comparing their impact on early and late events are available. In the present study, retrospective data on 351 implants in 266 routine patients inserted within a period of 10 years were classified into early and late DIF and compared to each other in regard of the patient’s gender, age, site of implantation, implant geometry, and patients’ systemic diseases.

Early DIF is associated with impaired bone healing and a reduced amount of implant primary stability by insufficient bone-to-implant contact [2, 5, 8, 9]. Factors like heat-induced necrosis and incorrect positioning may lead to impaired osseointegration [9, 16, 17] resulting in early implant loss. Furthermore, systemic factors influencing the patient’s wound healing capability [5, 15, 20, 26–29] and local inflammation could be associated with an early DIF as well [18, 25, 33]. Contrary, late DIF is defined by a reduction of implant stability after a latency of 6 months [5, 11, 12, 14]. This is thought to be a multifactorial process, as both implant and patient-related factors influence the implants’ long-term survival. On the one hand, loading distribution is affected by the implant’s geometry as well as the type of prosthetic treatment in particular different occlusion concepts [18–20]. On the other hand, local risk factors like plaque accumulation, gingivitis, bone quality and quantity, oral hygiene, periodontal disorders, and chronic occlusal trauma determine the implants’ long-term outcome [9, 21–25].

The choice of a cut-off point comprises methodological problems: Event-based cut-off points like abutment connection [34]/prothesis placement [35] might result in an underestimation of failures due to impaired osseointegration. For example, in one-stage surgery/immediate loading, there is no time at risk. On the other hand, a time-based cut-off point as reported by Antoun et al. [36] might result in an overestimation of early DIF [13].

As implant failure is more a fluent process than an event, all cut-off points comprise of imprecisions. In a large retrospective clinical study, Jemt differentiated between event-based and time-based cut-off points and found a higher number of implant failures in the “time-based” group (*n* = 81 implant failures at first year of follow-up vs. *n* = 73 implant failures at “prothesis placement” as event-based cut-off point). Furthermore, he reported only 5% of DIF after a period of 8 months. As an event-based cut-off point depends on the surgical protocol that might differ significantly between one patient and another, and a time-based cut-off point at first-year results in an overestimation of early DIF [13], this work set a cut-off point at 6 month after implant placement especially as osseointegration takes approximately 3 to 6 months (depending on implantation site and bone quality) [37, 38].

As described previously [39, 40], gender did not show any correlation to DIF within this study. Within the contingency analysis, a higher age was correlated to late events, whereas younger patients tend to lose implants earlier. This result corresponds with previous studies [40]. Lin et al. analyzed a total of 403 implants in an intermediate-term clinical study and detected a statistically significant OR for late implant loss for patients older than 40 years [14]. In case of younger patients, early DIF may occur more often as they are more prone to an active lifestyle together with a potentially earlier overload of the placed implants. For older patients, an etiologically correlation to the patients’ multi-morbidity, a decreased bone density, and several additional factors associated with age-related changes could be assumed [27, 41].

In the present study, early DIF was more frequent in implants placed in the mandible compared to the maxilla. As it impairs the process of osseointegration, thick and hard cortical bone and a reduced vascularization might affect the implant’s short-term prognosis. Furthermore, a significant higher rate of late DIF was seen in the maxilla when compared to the mandible. This is in accordance to the literature [40]. As the upper jaw consists of a wide-meshed cancellous bone, the respective bone density is lower when compared to the mandible. This, in combination with the proximity to the sinus resulting in a reduction of bone quantity [42, 43], may cause a lower long-term stability. As studies report contradictory findings regarding the long-term prognosis for implants inserted either within the anterior or posterior area of the jaw [13, 14], the present study gave evidence that posteriorly placed implants are in danger of DIF. Even if no statistically significant correlation was found, early and late DIF were more frequent in the posterior jaw (early DIF, 52.63%; late DIF, 12.77%) compared to the front tooth area (early DIF, 29.32%; late DIF, 5.26%). Impaired osseointegration resulting of thick bone and less blood supply, in combination with an occlusal overload within the early loading phase might explain these findings.

Despite current research, the effect of the implants’ geometry is a controversial issue. Several studies showed that small implants failed more often and especially earlier than larger implants [24, 44, 45], whereas others could not show any significant correlation between implant length and early or late events [46]. The present study showed implants of 10 mm in length to be positively correlated to both early and late DIF, whereas implants of bigger length were more likely to exhibit a late event than smaller implants. As the implants’ geometry affects the loading distribution, a correlation to late DIF is possible [18–20]. In contrast, there was no statistically significant correlation between the implant’s diameter and either early or late events within this study. Alsaadi et al. showed significantly higher late failure rates for implants of increasing diameter [24], whereas others recommended implants of wider diameter to decrease stress transferred to the surrounding bone [47]. In the study at hand, a heterogeneous patient collective was examined, reflecting daily praxis. Even so, this might have led to a relevant bias as it is possible that implants of larger diameter and length were predominantly inserted into the augmented bone of reduced quality resulting in an increased late DIF. Furthermore, it is possible that inserted implants of predominantly used geometric features led to higher failure rates in the respective groups.

Within this study, there was no statistically significant correlation between the occurrence of two systemic diseases and DIF even though cardiovascular diseases like hypertension are suspected to reduce bone density and healing capacity [48–51]. Furthermore, the respective medication might affect bone metabolism even if in vivo studies could not show any correlation [52–56]. Diabetes mellitus is known to induce xerostomia, caries, periodontitis and deregulation of immunity [57], infections and a reduced healing capacity [58, 59], microvascular changes [60], osteopenia with a 50% reduction of bone diameter and 30% less implant-bone contact [61, 62]. Despite a well-documented evidence of changes in metabolism, so far, no difference could be detected in human studies [4, 39, 63–66]. In accordance to the presented findings, Alsaadi et al. could not show an effect of cardiovascular diseases, diabetes mellitus, or thyroid dysfunction on dental implant failure [24].

## Conclusion

There is a lack of evidence-based information about potential risk factors for early and late DIF. However, the identification of these risk factors is crucial for creating individual treatment plans. An increased likelihood of early DIF was present in implants located in the mandible—especially in the posterior part of the jaw. Contrary to this, a higher patient age, a localization within the maxilla, and a greater implant length was associated with late DIF. However, neither systemic diseases nor the patient’s gender did show any correlation to implant failure. As risk factors for early and late DIF differ significantly—as shown within this study—an interdisciplinary evaluation and careful analysis is crucial to develop an individual therapeutic concept and to achieve the best possible result.

## Data Availability

The dataset supporting the conclusions of the article is included within the article and its additional files. The raw data analyzed during the current study is available from the corresponding author on reasonable request.

## Abbreviations

DIF: Dental implant failure
OR: Odds ratio
CI: Confidence interval
CVD: Cardiovascular diseases
